# Climate Change and Cardiovascular Health: Advancing Resilient Action Beyond COP30

**DOI:** 10.5334/gh.1550

**Published:** 2026-05-05

**Authors:** Poornima Prabhakaran, César Berenstein, Sarah Hamidi, Rajesh Vedanthan, Thomas Münzel

**Affiliations:** 1 Centre for Health Analytics Research and Trends, Trivedi School of Biosciences, Ashoka University, India; 2 Cardioecology and Healthy Habits Council, Argentine Society of Cardiology, Advisory Council, Interamerican Heart Foundation, Instituto Médico de la Comunidad, El Bolsón, República Argentina, Argentina; 3 World Heart Federation, Switzerland; 4 Institute for Excellence in Health Equity, NYU Grossman School of Medicine, New York, USA; 5 Department of Population Health, NYU Grossman School of Medicine, New York, USA; 6 University Medical Center Main, Department of Cardiology, Johannes Gutenberg University, Germany

**Keywords:** cardiovascular health, climate change, COP30

## Abstract

COP30 in Belém highlighted the urgent intersection of climate change, air pollution, and cardiovascular health. Climate hazards including heatwaves, floods, wildfires, and deteriorating air quality disproportionately affect vulnerable populations, exacerbating cardiovascular disease and straining fragile health systems. Air pollution, a major contributor to global CVD mortality, shares common sources with climate change, yet both act through distinct health pathways. Low- and middle-income countries face the greatest burden, reflecting inequities in social determinants, energy use, and urban infrastructure. The World Heart Federation emphasizes that climate action is cardiovascular action: reducing emissions, improving air quality, and building climate-resilient health systems are among the most effective preventive cardiology interventions. The Belém Health Action Plan provides a framework for integrating CVD into national climate strategies, strengthening health system preparedness, and fostering community resilience. Political leadership is critical to translate commitments into tangible health gains, as exemplified by successful urban interventions. Protecting hearts requires protecting the planet; coordinated climate-health action offers a pathway to healthier populations and sustainable societies.

## Introduction

COP30 in Belém marked an important milestone in advancing health within global climate discussions. Climate change manifests as myriad climate hazards, including heatwaves, droughts, wildfires, deteriorating air quality, floods and severe weather events. These impact access to safe food, clean air and drinking water, housing and healthcare, besides affecting education and livelihoods, with detrimental consequences to the overall health of affected populations (1). The escalating hazards in vulnerable regions with already fragile health systems can disrupt care, often leading to the worsening of existing morbidities, including cardiovascular illness. Yet, globally, risk determinants of a changing climate remain poorly addressed. Climate change and air pollution share their sources, such as the combustion of fossil fuels from industrial activity, transportation, and energy production. In rural areas, industrial agriculture and intensive livestock farming are responsible for releasing ammonia, a precursor of particulate matter and 30% of the GHGs (2). While they often arise from the same activities, air pollution and climate change affect health through different pathways. Importantly, air pollution is not primarily caused by climate change but by fossil fuel combustion, although climate change can worsen air pollution episodes, for example, through increased wildfires, higher temperatures that accelerate atmospheric chemical reactions, and stagnant air conditions.

Air pollution, an important upstream factor of climate change, has also emerged as one of the most significant risk factors for cardiovascular disease (CVD). Every year, 7.9 million deaths are attributed to air pollution (3), making it the second leading cause of mortality worldwide after high blood pressure (4). The World Health Organization (WHO) data likewise show that 6.7 million deaths are linked to combined ambient and household air pollution, with 62% from CVD, including over 2.6 million deaths from ischaemic heart disease and 1.5 million from stroke (5).

The World Heart Federation (WHF) has long highlighted the critical link between air pollution and cardiovascular health, advocating for stronger integration of clean air policies into global health and development agendas. Yet, air pollution is only one dimension of the broader climate–cardiovascular nexus. Compounding pressures from co-existing climate hazards can lead to worsening illness and even death in those with already existing diseases (6). Synergistic effects have been demonstrated, for example, between high temperatures and air pollution, producing higher risks for cardiovascular death than either exposure alone.

The burden is most significant in low- and middle-income countries, where reliance on fossil fuels for electricity, polluting fuels and technologies at the household level, and rapidly urbanizing cities with unclean energy systems disproportionately harm the poorest communities (7), even though historically, these countries are generally not the highest global emitters of fossil fuels. For them, polluted air and rising heat are daily realities that drive health inequities and strain fragile health systems. Evidence has shown that vulnerability to climate change-related adverse health outcomes, including CVD, is largely driven by the uneven distribution of social determinants of health, such as poverty, housing quality, urban infrastructure, and access to healthcare (8). Health systems that are under-prepared, ill-equipped and already overburdened with delivering existing healthcare to their people will further suffer from disproportionately greater impacts due to worsening cardiovascular health and disrupted care during extreme climate events and poor air quality.

Despite this, the intersection of climate and health, including cardiovascular health, remains underrepresented in global climate negotiations, too often confined to side events or ‘health days’ at COP. Yet the reality is clear: climate action is cardiovascular action and prevention. Reducing emissions, improving air quality, and building climate-resilient and low-carbon health systems are the most effective preventive cardiology measures available at the population level (9).

As the global voice of the cardiovascular community, WHF reaffirms its commitment following COP30: to protect hearts, we must protect the planet. CVDs must be at the center of the health narrative in climate discussions. Integrating cardiovascular health into climate policy is not only a moral imperative; it is a pragmatic pathway toward healthier people and a sustainable planet.

### I. Climate Stressors and CVD: A Converging Crisis

Climate change contributes to the development of non-communicable diseases, mainly CVD. There are direct and indirect ways in which climate change can produce disease or aggravate cardiovascular health, leading to premature deaths. Direct pathways include rising temperatures and deteriorating air quality that increase the risk of CVD inducing systemic inflammation, endothelial dysfunction and autonomic imbalance. Additionally, dehydration associated with extreme heat can cause low blood volume and increased blood viscosity, which are factors that expose vulnerable individuals to acute cardiovascular events (10).

Indirectly, extreme weather events like floods, droughts, and wildfires, which induce stress and vascular inflammation, increase the probability of cardiovascular events. Another effect of rising temperature is the climate penalty. It means the formation of secondary pollutants, such as ground-level ozone, under altered meteorological conditions associated with hot weather (11). Ozone functions both as a short-lived climate pollutant and an air pollutant; its health impacts are primarily on the respiratory system, though it contributes to overall air pollution exposure (12). At the same time, fine particulate matter (particularly PM2.5) remains the air pollutant with the strongest evidence for cardiovascular harm. PM2.5 particles are small enough to deposit deep in the alveoli of the lungs and can penetrate into the circulatory system, carrying reactive surface chemicals throughout the body (10,13).

Climate change is, therefore, not a distant threat. Whether acting alone or in concert with air pollution, it is now a pivotal environmental determinant of circulatory and cardiovascular health. WHF continues to work to translate science into policy, urging governments, agencies, and partners to take coordinated action to mitigate climate impacts and reduce global cardiovascular risk.

### II. Why CVD as a Lens for Climate Action?

There are numerous interventions that can have co-beneficial effects on cardiovascular health and planetary health (9). These include shifting the balance from red meat-based diets to plant-based diets (14), expanding green spaces in residential and urban environments (15), transitioning from vehicular to more physically active forms of transportation (16), increasing the use of clean/renewable sources of electricity (17), using cleaner stoves and power sources for cooking/heating (18), and practicing resource efficiency in health care (19). Urban planning measures, such as avoiding mixed residential–industrial zoning and designing 15-minute neighborhoods, can further reduce pollution exposure, enhance active mobility, and improve access to essential services (20), thereby supporting cardiovascular health. Several of these initiatives can also increase productivity and economic output, reduce poverty, and generate other societal benefits (21). Finally, actions to improve cardiovascular health can also have ‘positive spillover’ effects on overall health outcomes, particularly for other non-communicable diseases and chronic infectious illnesses. Thus, there can be ‘quadruple co-benefits’ of climate action when initially centered on improving cardiovascular health (22).

### III. Building Forward: From COP30 to Coordinated Implementation

Previous COP conferences centered on negotiations and national decisions with limited attention in the interim periods on decentralized implementation and action on the ground in member countries. Following COP30 in Belém, widely stated to be the ‘Implementation COP’, it is apt to shift the narrative to global adaptation practices and implementation. The next phase must therefore ensure that climate commitments translate into real-world climate action with tangible health benefits, particularly for CVD. Strengthening national implementation, supporting resilient health systems, and enhancing community preparedness offers a pathway to ‘heart-resilient’ societies, where protecting the climate directly safeguards cardiovascular health.

The Belém Health Action Plan (BHAP) (23), launched on November 13, 2025, provides a practical blueprint to move from evidence to coordinated action at the national and community levels. With a strong emphasis on adaptation to protect populations from climate-related health risks, the BHAP builds on the recognition that climate change and health are deeply interconnected and outlines priority measures for governments and partners to operationalize this agenda. WHF provides recommended actions aligned with the BHAP below:

1. **BHAP 1.2**. Identify a Priority List of Climate-Related Risks and Diseases and Develop Strategies to Address Them:
  RECOMMENDED ACTION: Country-level risk assessments of disease burden can inform the development of national health action plans that integrate CVD into climate and air quality strategies. The WHO 2021 air quality guidelines provide clear steps to mitigate air pollution at source, including from the combustion of fossil fuels.
  Way forward: National action plans – Every government should integrate cardiovascular health into its national climate, air quality, heat, and adaptation strategies. Embedding cardiovascular indicators within Nationally Determined Contributions (NDCs) and National Adaptation Plans (NAPs) can deliver measurable health co-benefits while advancing environmental goals. Aligning air quality standards with WHO air quality guidelines and accelerating fossil fuel phase-out will protect both planetary and cardiovascular health.
2. **BHAP 1.3**. Strengthen Preparedness and Strategic Stockpiles of Supplies, Vaccines, and Medicines
  RECOMMENDED ACTION: Establishing and strengthening resilient health systems that ensure appropriate preparedness plans to adapt to climate shocks and deliver uninterrupted care for NCDs through regular planned stock-taking of medicines and diagnostics.
3. **BHAP 2.2**. Smart Workforce to Manage Climate Change Challenges
  RECOMMENDED ACTION: Capacity-building and sensitization of the health workforce with enhanced focus on the community of cardiologists, physicians, and medical students to screen, treat and prevent a surge in climate-related illnesses, including CVDs, by recognizing the risk factors and advocating for measures to address them.
  Way forward: Resilient health system – Climate shocks, heatwaves, floods, and dust storms disrupt access to chronic disease care and threaten continuity of care. Health systems must plan for continuity of CVD care, from temperature-controlled drug storage to backup power for hospitals and telemedicine links during disasters. Training a ‘climate-smart’ health workforce will be critical; clinicians, nurses, and emergency teams must be prepared to manage climate- and/or pollution-sensitive cardiovascular conditions such as heat-induced arrhythmias, dehydration, and myocardial infarction.
4. **BHAP 2.3**. Promote Community Resilience and Climate Awareness
  RECOMMENDED ACTION: Decentralizing climate awareness to strengthen community resilience and promote coherent response mechanisms for NCD care during climate shocks will translate to improved healthcare access, care delivery, and health outcomes, especially for the most vulnerable.
5. **BHAP 2.5**. Multisector Strategies for Public Policy with Health Co-Benefits
  RECOMMENDED ACTION: Engage communities, urban planners, meteorological departments, energy, transport, agriculture and food systems, waste and sanitation departments, and manufacturers and suppliers of goods and services for a coordinated response to climate stressors and to protect overall health.
  Way forward. Community resilience and intersectoral coordination – Effective climate health action extends beyond hospitals. It requires coordination with urban planners, transport authorities, and the energy sector to build healthier, cooler, and cleaner environments. Early warning systems, green public spaces, and active mobility policies can simultaneously reduce exposure, emissions, and disease risk.

### IV. The Political Imperative: Transforming Climate Action into Cardiovascular Protection

Climate action can only reach its full potential when health is recognized as a foundational pillar. Despite decades of warnings, the world’s largest emitters continue to subsidize fossil fuels, expand extraction, and delay decisive action, while millions die each year from pollution-driven cardiovascular disease.

The time for polite appeals has passed. After COP30, it is undeniable that the greatest barriers to protecting global cardiovascular health are often political, not scientific. We know the causes. We know the solutions. We even have implementation frameworks such as the Belém Health Action Plan. What is often missing is the political will to address sources such as the industries and interests that profit from pollution, fossil fuels, and environmental degradation. Every fraction of a degree of warming and every microgram of particulate matter translates into higher blood pressure, more heart attacks, and shortened lives, making climate policy that fails to curb emissions and pollution, in effect, a policy that accepts preventable death.

Political inaction, however, is not inevitable. Where decisive governance has been exercised, climate action has delivered rapid and measurable cardiovascular health gains. In Barcelona, for example, targeted urban policies introduced since 2013 to green public spaces, reduce vehicle congestion, and improve air quality led to a 30% reduction in nitrogen dioxide concentrations within eight years. Measures included the expansion of public and active transport networks, the introduction of a low-emission zone, and the electrification of municipal vehicle fleets, reducing the most polluting journeys while increasing walking and cycling and demonstrating that political leadership can translate climate ambition into tangible health outcomes (10).

Leaders must therefore act not out of political convenience but moral responsibility because clean air, climate mitigation, and adaptation to a changing climate and its related hazards are the most powerful cardiovascular interventions of our time, and anything less is indefensible. Moving from commitments to implementation therefore requires decisive action across all sectors:

**CALL TO ACTION**

- **To governments and negotiators:** Put cardiovascular health at the center of climate and clean air action; enforce WHO 2021 air quality guidelines, embed CVD in NAPs, and advance decisive action against fossil fuels in a fair and equitable manner to prevent avoidable deaths.
- **To health systems:** strengthen climate-resilient care pathways and guarantee uninterrupted NCD/CVD services during heatwaves, floods, and other climate shocks, including air pollution episodes. Train health workers to respond to climate- and air pollution-related cardiovascular risks. Build capacity across the workforce to integrate climate-health considerations into routine care.
- **To the cardiology community:** Treat climate stressors as major upstream CVD determinants and advocate forcefully for clean air, emission reduction, and resilient environments.
- **To civil society:** Exert sustained pressure for climate action, framing it through its immediate cardiovascular and health impacts and by advocating for stronger community climate resilience measures.

COP30 underscored that protecting the planet and hearts are inseparable goals. WHF stands ready to lead and support the global cardiovascular community in ensuring climate action translates into greater climate resilience, healthier hearts, and healthier societies.
